# Increased eating disorder frequency and body image disturbance among fashion models due to intense environmental pressure: a content analysis

**DOI:** 10.3389/fpsyt.2024.1360962

**Published:** 2024-04-03

**Authors:** Nikolett Bogár, Pál Kővágó, Ferenc Túry

**Affiliations:** ^1^Semmelweis University, Institute of Behavioural Sciences, Budapest, Hungary; ^2^Pázmány Péter Catholic University, Institute of Psychology, Budapest, Hungary

**Keywords:** anorexia nervosa, appearance pressure, body image disorder, content analysis, eating disorders, fashion industry, fashion models

## Abstract

**Introduction:**

Female fashion models are under intense occupational pressure. The present study focuses on assessing the lived experience of fashion models with regards to their dieting and exercising habits, body image perception, eating disorder-like symptoms, and experience of abuse via self-narrated reports.

**Methods:**

Series of open questions were distributed among international fashion models (N=84, mean age=23.2 years; mean BMI=16.9) selected by convenience and snowball sampling. Models from 17 countries participated. The questions targeted models’ eating, exercising, dieting habits, body image perception, and eating disorder symptoms. The average word count of the transcripts was 2473.9 (SD = 2791.6). Thematic content analysis was performed on the transcripts. A total of 31 codes were created to address disordered eating and body image concerns.

**Results:**

Negative body-related claims appeared in 89.3%, and positive claims in 64.3% of the models’ transcripts. Negative remarks about eating were made by 45.2% of the participants, and 23.8% positively. Control over their food intake was exercised by 78.6% of the participants and 40.5% used extreme calorie restriction. Models who talked more positively about their bodies expressed significantly more frequently extreme calorie restriction. Extreme sports habits occurred in 23.8% of the transcripts, obsessive sports habits were claimed by 11.9% of participants. Self-induced vomiting was prominent in 14.3% of the answers. Criticism from other industry members was experienced by 83.3% of the participants while 44.0% received body appreciation. Body image disorder-like symptoms were expressed by 63.1% of models. Such models mentioned significantly more often content about eating disorders and talked significantly more negatively about eating. Psychological problems were mentioned by 48.8%, whereas 16.7% took part in psychotherapy. Those who partake in therapy mentioned significantly more eating disorder content in their narratives. Abuse was mentioned by 25.0% of the models.

**Conclusion:**

Fashion models are experiencing increased environmental pressure to conform to the extreme slimness ideal. There is a heightened prevalence of disordered eating and other weight-controlling behaviours among fashion models to succeed in their careers. Qualitative research is crucial in understanding the more subtle dynamics in conforming to and maintaining the thin beauty ideal.

## 1 Introduction

Data on the prevalence of disturbed eating habits and body image concerns among fashion models is scarce. This can be due to the closed structure of the unique profession and the concern over potential repercussions stemming from disclosure of confidentialities (1, 2). However, with the rise of unrealistic beauty standards (3) and the increasing rates of eating disorders (EDs) (4) it is more important than ever to investigate this specific group.

Professions that necessitate a low body weight and are associated with appearance, such as ballet dancers, marathon runners, and flight attendants, are more prone to an increased risk of developing EDs (5). Models are facing intense pressure to reach and to maintain the size requirements dictated by the fashion industry (6). Contrary to the pursuit of bigger size representations, the beauty standards remained similar in the past decade: height of at least 175 cm, waist circumference around 60 cm and hip circumference preferably not bigger than 90 cm (7, 8). Even though regulations have been implemented on several international fashion markets (9), the majority of models appearing on the runways, e.g. on New York Fashion Week, still has a BMI under 18.5 (10), which is considered underweight (11). These unrealistic measurement requirements put sociocultural pressure on young women, both models and non-models, to conform to the thin beauty ideal (12). According to Rodgers et al. (13), 62.4% of female fashion models have been asked to lose weight or to change their body shape and/or size. Presumably due to the intense appearance pressure, models are at significantly greater risk for developing subclinical anorexia nervosa (AN) compared to non-models (14).

Peer-pressure, the abundantly displayed strong emphasis on thin body ideals, appearance related criticism are risk factors for body image concerns and EDs (15). Body dissatisfaction foresees the emergence of EDs of clinical severity (16). In the fashion industry, models are often encouraged by their agents to lose weight, even though they are already thin (17). In a study, 63.1% of models reported that they would receive more job offers providing that they were slimmer (13). Weight loss remains a pivotal element and a major accomplishment in the fashion industry (18). Such criticism can highly influence body perception, self-esteem, and cause the feeling of shame which is correlated with disordered eating habits (19).

Besides the considerable mental health consequences, the lack of proper nutrition can cause several somatic symptoms, such as osteoporosis, cardiac complications, brain shrinkage or amenorrhoea (20–22).

The difficulties of partaking in modelling extend beyond eating and body image disturbances. The presence of abusive or traumatic experience is not unusual (18). This might further enhance the risk for the development of EDs, as both traumatic events and the lack of psychosocial resources are associated with higher ED frequency (23).

The present study aimed to explore the nuanced relationship between fashion models and their dietary habits, exercise routines, body image perception, ED-like symptoms, experience of abusive nature amongst other factors relevant in the scope of ED development in the sociocultural context of the fashion industry via self-narrated reports. We trust that this qualitative research adds great value to the limited number of existent quantitative findings, and to better understand lived experience of models. There exists a significant gap in the literature in the assessment of subjective experiences influencing ED risk factors. To our knowledge, amongst all the research conducted about fashion models, only one study has previously applied qualitative evaluation, specifically interpretative phenomenological analysis (18). Our research aims to fill this gap, enriching the understanding of the sociocultural dynamics at play and providing a grounded perspective on the personal and professional challenges models face, and contributing to targeted interventions and policy recommendations within the industry.

## 2 Materials and methods

### 2.1 Measures

A series of open questions were distributed among fashion models, aiming to gather information about their careers, their view on the fashion industry, their relationship with their agents, and their attitudes towards their body image, eating, exercising and dieting habits. The list of questions contained 23 open questions concerning their health, diets, the requirements enforced by the industry, and it also touched on their self-perception including insecurities about their looks, and all questions were registered at one occasion. Data was collected about their age, their experience in the fashion industry, their nationality, height and weight. The answers to the series of open questions were gathered via e-mail (N = 73, in a Microsoft Word format) and online video calls (N = 14). Participants were provided the possibility to answer all questions in free text or free speech, enabling them to fully elaborate on their experience (see **Supplementary Material 1**). All participants received the same questions. The online calls were recorded, and their transcripts were created within 24 hours to be analyzed in the same format as the written answers.

### 2.2 Sample

Data was collected from 87 participants who were recruited with convenience and snowball sampling method (the first author worked in the fashion industry as a model for five years). The data collection took part between 2016 June and 2021 May. Three participants were omitted from the analysis, since one of them was not an active fashion model, while two participants’ interviews were not recorded in their entirety. In total, 84 transcripts were analyzed. The mean age of the participants was 23.23 years (SD = 4.4, range 16 – 34 years). Their average experience in the fashion industry was 6.56 years (SD = 3.67, range 1 – 15 years), and their average BMI was 16.9 (SD= 1.60, range 14 – 23.7). It is remarkable that 36.4% of the models reported BMI of between 17.0 and 18.5, and a further 52.3% under 17.0 (moderately or severely underweight). Mean height of the fashion models was 177.8 cm (SD= 3.6, range 171–186 cm). Models of various nationalities participated in the study, from 17 countries, including American, Canadian, Dutch, English, French, Hungarian, Polish, Russian, and Spanish subjects, amongst others. The average wordcount of the transcripts was 2473.9 (SD = 2791.6).

### 2.3 Thematic content analysis

Before starting the coding procedure, all transcripts was fully anonymized, and identifiers were omitted. The collected data was analyzed by performing a thematic content analysis (24). After the data collection phase, the video call interviews were read and transcribed. After the critical reading of the transcripts a coding booklet was developed containing coding instructions on 31 codes. This coding booklet was developed by the authors of the present paper. The codes were in alignment with our research questions such as attitudes towards one’s body, weight and exercise. Other codes referred to symptoms of EDs according to DSM-5-TR, since the goal of our research was to connect attitudes to various ED-like symptoms. The codes used in the current analysis are presented in **Table 1**. Before manually starting the coding procedure, all transcripts were fully anonymized, and identifiers were omitted. Only explicit mentioning of each content category was coded, possible latent contents were ignored in this analysis for better reliability. One testimony could receive multiple of the same codes, even contradictory ones (e.g., positive and negative attitudes towards their bodies within one testimony). The level of analysis was semi-sentences.

**Table 1:** Content analysis codes with their brief description, the Krippendorff’s alpha values of the two independent judges’ coding and their occurrence amongst fashion model subjects (N=84).

Code name (Krippendorff’s alpha)	Code description	Occurring statements (Number of models; %)
**Weight** (Positive:.545; Negative:.551; Neutral:.499)	Every mention of the participant’s weight.	Positive: 17; 20.2% Negative: 46; 54.8% Neutral: 66; 78.6%
**Exercise, training, sport** (Positive:.549; Negative:.732; Neutral:.746)	Every mention of the participant’s exercise, training, or sports habits.	Positive: 34; 40.5%; Negative: 12; 14.3%; Neutral: 77; 91.7%
**Body** (Positive:.547; Negative:.541; Neutral:.517)	Every mention of the participant’s body perception.	Positive: 54; 64.3%; Negative: 75; 89.3%; Neutral: 80; 95.2%
**Eating** (Positive:.635; Negative:.618; Neutral:.391)	Every mention of the participant’s experiences, habits with eating.	Positive: 20; 23.8%; Negative: 38; 45.2%; Neutral: 81; 96.4%
**Body appreciation** (.664)	Every mention where the participant expresses receiving appreciation toward their body from other industry members.	37; 44.0%
**Body critique** (.634)	Every mention where the participant expresses receiving criticism toward their body from other industry members.	70; 83.3%
**Abuse** (.259)	Every explicit mention of abuse (verbal, physical, emotional, or other) suffered by the participant.	21; 25.0%
**Monotrophic diet** (.391)	Every explicit mention of a type of diet that involves eating only one food item.	23; 27.4%
**Extreme calorie restriction** (.487)	Every explicit mention of extreme calorie restriction.	34; 40.5%
**Dietary control** (.618)	Every explicit mention of the participant’s will to greatly control their eating habits.	66; 78.6%
**Loss of dietary control** (.667)	Every explicit mention where the participant admits they lost control of their eating habits.	19; 22.6%
**Liquid diet** (.75)	Every explicit mention of the participant’s liquid diet.	3; 3.6%
**Overeating and binge eating episodes** (.628)	Every explicit mention that the participant has significantly overeaten or had a binge eating episode.	14; 16.7%
**Self-induced vomiting, purging** (.731)	Every explicit mention that the participant has purged themselves by self-induced vomiting after eating.	12; 14.3%
**Extreme sports habits** (.532)	Every explicit mention that the participant has got extreme sporting habits.	20; 23.8%
**Obsessive and/or compulsive sporting habits** (.456)	Every explicit mention that the participant has got obsessive and/or compulsive sports habits.	10; 11.9%
**Consumption of laxatives** (.896)	Every explicit mention that the participant consumes laxatives without a specific medical reason.	6; 7.1%
**Fear of gaining weight** (.343)	Every explicit mention that the participant has got heightened fear from gaining weight.	18; 21.4%
**Lack of or irregular menstruation cycle** (.749)	Every explicit mention that the participant experiences lack of or irregular menstruation cycle.	16; 19.0%
**Body image disorder** (.375)	Explicit signs of body image disorder.	53; 63.1%
**Eating disorder** (.567)	Explicit signs of eating disorder.	31; 36.9%
**Psychological disorders** (.42)	Every explicit sign of psychological disorders.	41; 48.8%
**Therapy** (.698)	Every explicit mention that the participant underwent or is currently treated in psychotherapy.	14; 16.7%

Positive or negative valence was added to a code if the participant’s attitude toward the subject is clearly stated. Neutral code was added if the attitude is not clearly mentioned. The valence is added to each individual occurrence.

Coders were recruited from Pázmány Péter Catholic University, Budapest, Hungary who attended a content analysis course during their psychology MSc programs. They received no benefits for their work, and they volunteered to join the coding process. They had knowledge that they participate in research focusing on fashion models’ lived experiences, however they were not introduced to the research goals. This means that while they were aware of the codes they were supposed to locate, they had no information as to what kind of statistical analyses were to be performed after their work. The Coders were trained to use the coding booklet. A sample was sent out to the coders for training purposes. The texts were broken into semi-sentences and sent to the coders in a spreadsheet where each line of the first column contained one semi-sentence, and every column corresponded to one code. Coders needed to mark line by line whether it contained the specific code. The coding was later evaluated by the second author who is an experienced researcher in content analysis. Coding inaccuracies were assessed, discussed, corrected and coders were re-trained before receiving more transcripts. The testimonies were assigned randomly to the coders, and they were hand-coded by at least two people. Krippendorff’s alphas were calculated using Hayes and Krippendorff’s (25) KALPHA algorithm (see **Table 1**). Every coding difference was sorted with the involvement of a third judge with experiences in eating and body image disorders, who only coded the parts where the two judges’ interpretation differed. Due to the large fluctuations in the wordcounts, the analyses were conducted using relative frequencies. The absolute frequency of the codes was divided by the wordcount corresponding to the transcripts.

### 2.4 Data analysis

The coding process’ resulted in a table containing the frequency of the codes per transcript. Data analysis was performed on two levels. Firstly, frequencies of each code were calculated and transformed into percentages to determine what rate each code appears in all of the transcripts. Secondly, relative frequencies were calculated: each code’s frequency divided by the corresponding transcript’s wordcount. These relative frequencies allowed for the comparison of the transcripts. Mann-Whitney U tests were performed using the relative frequencies of the codes as dependent variables, while grouping the transcripts along various codes for ex. transcripts with or without signs of overeating.

### 2.5 Ethical approval

The research is in accordance with the Helsinki Declaration and was approved by the Regional Research Ethical Board of the Semmelweis University Budapest (No. 3/2020). Written informed consent was obtained from all participants included in this study.

## 3 Results

**Table 1** contains the number of models and the percentage of the prevalence of each code. The distribution of the codes was analyzed, and non-parametric Mann-Whitney U tests were performed using the relative frequencies for each code for a more in-depth analysis. Due to the extensive number of possible relations between each codes, only the significant relations will be demonstrated.

### 3.1 Body related statements

The mostly referenced code in the current study was statements about the subjects’ body. Negative claims appeared in 89.3% of the models (‘I felt fat and hated it’) while 64.3% mentioned positive remarks about their bodies (‘I think that my body is perfect’). Models who talk positively about their bodies also mention significantly more frequently extreme calorie restriction (*U* (*N*_posbody_=14, *N*_negbody_= 70) = 661, *z* = 2.310, *p* = .021) and monotrophic eating (*U* (*N*_posbody_=14, *N*_negbody_= 70) = 613, *z* = 1.879, *p* = .041). These individuals talked significantly more about body image disorder-like symptoms (*U* (*N*_posbody_=14, *N*_negbody_= 70) = 715, *z* = 2.771, *p* = .006) and psychological disturbances (*U* (*N*_posbody_=14, *N*_negbody_= 70) = 673, *z* = 2.360, *p* = .018). Participants who talked positively about their bodies made significantly more negative statements about their weight (*U* (*N*_posbody_=14, *N*_negbody_=70) = 682.5, *z* = 2.425, *p* = .015). Neutral statements were made by 95.2% of the models, such as ‘*Hmmm, my measurements. I’m 184 cm tall and 48-49 kg’*. Those participants mentioned significantly more positive statements about their bodies who did not talk about body image disorder-like symptoms (*U* (*N*_nobodyimagedisorder_=31, *N*_bodyimagedisorder_=53) = 611.5, *z* = -1.992, *p* = .001).

More than half of the participants, 54.8% had negative opinion about their weight (‘I was desperate to lose weight’), while only 20.2% of the models said positive affirmations in the same regard (‘I’m feeling so comfortable, with my body, with my weight’). Neutral statements of their weight (‘I’m 54 kg.’) were made by 78.6% of the models – most likely due to the nature of the questions. Negative weight related statements were significantly more frequent among models who show body image disorder-like symptoms (*U* (*N*_nobodyimagedisorder_=31, *N*_bodyimagedisorder_=53) = 1151, *z* = 3.206, *p* = .001).

### 3.2 Eating related statements

Nearly half of the participants (45.2%) said something negative about eating habits or their attitudes towards eating, e.g., ‘I couldn’t enjoy food anymore in a normal way’. Positive opinion about eating (‘I love food and I love cooking.’) were given in 23.8% of the transcripts. The models who talked negatively about eating mentioned significantly more body image disorder-like symptoms (*U* (*N*_nobodyimagedisorder_=31, *N*_bodyimagedisorder_=53) = 1071, *z* = 2.530, *p* = .011). Those who talked more negatively about eating, talked significantly more often about overeating (*U* (*N*_noovereating_=70, *N*_overeating_=14) = 710, *z* = 2.888, *p* = .004). More than two thirds, 78.6% of the participants, have mentioned that they control their food intake, e.g., ‘[ … ] skipping lunch at school, skipping breakfast, or eat a few slices of apple or crackers’. Monotrophic eating (‘I started to lose weight by eating 3 apples a day’) occurred in 27.4% of the answers, and 40.5% claimed to have used extreme calorie restriction (‘I completely started starving myself. I was like on one apple a day pretty much.’) through their modeling career. Juice fasting was occurrent amongst 3.6% of the participants. According to the answers, 22.6% of participants have lost control over their food intake. These individuals portrayed significantly more frequent binge eating (*U* (*N*_lossofcontrol_=19, *N*_nolossofcontrol_=65) = 1033, *z* = 6.844, *p* <.000), self-induced vomiting (*U* = 874, *z* = 4.507, *p* <.000), extreme caloric restriction (*U* (*N*_lossofcontrol_=19, *N*_nolossofcontrol_=65) = 888, *z* = 3.256, *p* = .001), and obsessive sports habits (*U* (*N*_lossofcontrol_=19, *N*_nolossofcontrol_=65) = 770.5, *z* = 2.908, *p* = .004). They made significantly less positive remarks about their bodies (*U* (*N*_lossofcontrol_=19, *N*_nolossofcontrol_=65) = 435.5, *z* = -1.992, *p* = .046), more negative remarks about eating (*U* (*N*_lossofcontrol_=19, *N*_nolossofcontrol_=65) = 983, *z* = 4.274, *p* <.000), more frequent controlling of food intake (*U* (*N*_lossofcontrol_=19, *N*_nolossofcontrol_=65) = 865.5, *z* = 2.665, *p* = .008) and monotrophic eating (*U* (*N*_lossofcontrol_=19, *N*_nolossofcontrol_=65) = 857, *z* = 3.260, *p* = .001). Moreover, ED related statements are also significantly more frequent amongst these models (*U* (*N*_lossofcontrol_=19, *N*_nolossofcontrol_=65) = 1018, *z* = 4.948, *p* <.000) and they engaged more frequently in psychotherapy (*U* (*N*_lossofcontrol_=19, *N*_nolossofcontrol_=65) = 856, *z* = 3.928, *p* <.000). Binge eating was prominent amongst 16.7% of the models. Models who stated binge eating talked significantly more frequently about extreme caloric restriction (*U* (*N*_noovereating_=70, *N*_overeating_=14) = 674.5, *z* = 2.493, *p* = .013), self-induced vomiting (*U* (*N*_noovereating_=70, *N*_overeating_=14) = 739, *z* = 4.911, *p* <.000), obsessive exercising (*U* (*N*_noovereating_=70, *N*_overeating_=14) = 626.5, *z* = 2.913, *p* = .004), weight gain (*U* (*N*_noovereating_=70, *N*_overeating_=14) = 692, *z* = 3.378, *p* = .001) and ED symptoms (*U* (*N*_noovereating_=70, *N*_overeating_=14) = 751, *z* = 3.620, *p* <.000).

### 3.3 Sports habits related statements

**Figure 1:**
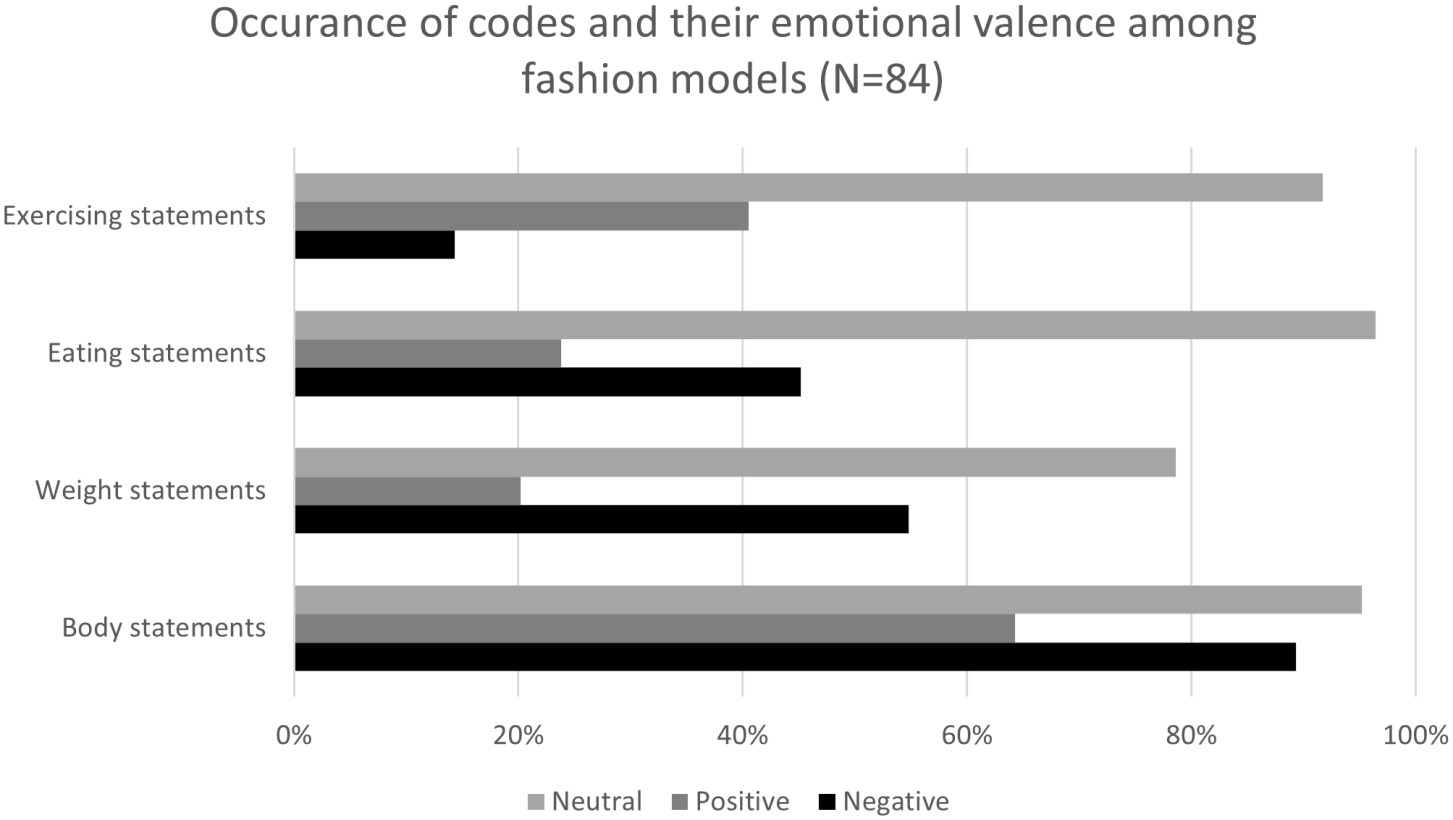
Occurrence of codes and their emotional valence among fashion models (N=84).

Statements about exercising were predominantly positive (‘I love doing Pilates’) or neutral (‘I work out 6 times a week’) (40.5% and 91.7%, respectively), and negative in only 14.3% of the cases (I didn’t feel motivated to workout’). Extreme sports habits (‘I worked out every day for 2 hours’) were portrayed by 23.8% of the participants. Obsessive sports habits (‘I wouldn’t leave until I would burn a specific number of calories’) were claimed by 11.9% of the participants. The occurrence of codes and their emotional valence is presented in **Figure 1**.

### 3.4 Purging related statements

Self-induced vomiting was prominent in 14.3% of the transcripts and it appeared significantly more frequently among models who experienced body image disorder-like symptoms (*U* (*N*_nobodyimagedisorder_=31, *N*_bodyimagedisorder_=53) = 1007.5, *z* = 2.833, *p* = .005). Laxative abuse was reported by 7.1% of the models and was mentioned significantly more by those individuals who talked about body image disorder-like symptoms (*U* (*N*_nobodyimagedisorder_=31, *N*_bodyimagedisorder_=53) = 914.5, *z* = 1.931, *p* = .050). None of the respondents who made positive statements about their bodies mentioned the usage of laxative substances.

### 3.5 Statements about external feedback on body

Our analysis shows that 83.3% of the participants were criticized by other industry members (‘My agent told me that I’m ugly’), while 44.0% received body appreciating affirmations (‘The sicker I was the more approval I was getting’). Models who talked more frequently about external criticism mentioned more body image disordered-like symptoms (*U* (*N*_nocrit_=14, *N*_crit_=70) = 637, *z* = 1.810, *p* = .050). The participants who experienced body appreciation talk more positively about their body (*U* (*N*_noappr_=47, *N*_appr_=37) = 1033, *z* = 2.099, *p* = .036). However, those individuals also talk more about self-induced vomiting (*U* (*N*_noappr_=47, *N*_appr_=37) = 1059.5, *z* = 2.813, *p* = .05), laxative use (*U* (*N*_noappr_=47, *N*_appr_=37) = 966, *z* = 1.947, *p* = .049), and engagement in psychotherapy (*U* (*N*_noappr_=47, *N*_appr_=37) = 1025.5, *z* = 2.165, *p* = .030).

### 3.6 Psychological disturbances related statements

**Figure 2:**
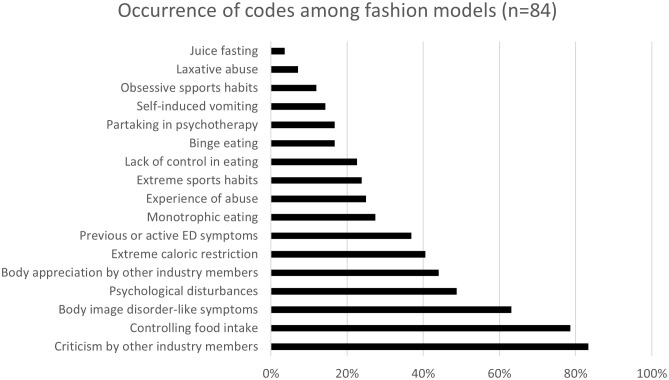
Occurrence of codes among fashion models (N=84).

Body image disorder-like symptoms (‘Even when I lost the weight to 45 kgs [I’m 5’11”] I still thought that I was fat’) were expressed by 63.1% of the participants. Significantly more negative remarks about one’s body were observed among participants who reported body image disorder-like symptoms (*U* (*N*_nobodyimagedisorder_=31, *N*_bodyimagedisorder_=53) = 1165.5, *z* = 3.186, *p* = .001). Models who reported body image disorder-like symptoms talked significantly more often about extreme caloric restriction (*U* (*N*_nobodyimagedisorder_=31, *N*_bodyimagedisorder_=53) = 1091, *z* = 2.812, *p* = .005), monotrophic eating (*U* (*N*_nobodyimagedisorder_=31, *N*_bodyimagedisorder_=53) = 1083, *z* = 3.086, *p* = .002), and losing control (*U* (*N*_nobodyimagedisorder_=31, *N*_bodyimagedisorder_=53) = 978, *z* = 1.980, *p* = .048). Previous, or active EDs were mentioned by 36.9% of the models. Those models who mentioned body image disorder-like symptoms mentioned significantly more often content about EDs as well (*U* (*N*_nobodyimagedisorder_=31, *N*_bodyimagedisorder_=53) = 1117, *z* = 3.165, *p* = .002). Psychological problems of different sorts (e.g., anxiety, depression, panic attacks, suicidal attempts) were mentioned by 48.8% of the models, while 16.7% confessed to taking part in psychotherapy. Those who engage in psychotherapy make significantly more remarks about losing control in eating (*U* (*N*_nother_=70, *N*_ther_=14)= 723, *z* = 3.817, *p* <.001), and talk significantly more about overeating (*U* (*N*_nother_=70, *N*_ther_=14) = 734, *z* = 4.512, *p* <.001) and self-induced vomiting (*U* (*N*_nother_=70, *N*_ther_=14) = 779, *z* = 5.710, *p* <.001). Furthermore, those who mentioned taking part in therapy talk significantly more about weight gain (*U* (*N*_nother_=70, *N*_ther_=14) = 694, *z* = 3.412, *p* <.001) and mention significantly more ED content (*U* (*N*_nother_=70, *N*_ther_=14)= 699, *z* = 2.899, *p* = .004) in their narratives. Those who do not engage in psychotherapy make significantly more negative remarks about their bodies (*U* (*N*_nother_=70, *N*_ther_=14) = 229, *z* = -3.135, *p* = .002). One quarter of the models (25%) mentioned statements referring to abuse (every content which refers to being subjected to physical or psychological violence). The occurrence of codes is presented in **Figure 2**.

## 4 Discussion

This study was designed with the intention to better understand the lived experience of fashion models with special regards to their eating and exercising habits, self-perception, potentially prevailing psychological disturbances, abuse, and other strongly related factors. The present study greatly adds to the scarce quantitative data about fashion models.

Fashion models encounter elevated levels of appearance and sociocultural pressure linked to symptoms of disordered eating. This pressure emanates from the prevailing extremely thin aesthetic in the fashion industry and is exerted by fellow members of the field (13, 26). The pressure to maintain an extremely slender physique, driven by professional standards and industry expectations, coupled with unrealistic ideals perpetuated by women seeking success as fashion models, is proposed to contribute to a more negative body image within this group (17). Up to this day, fashion runways are still overpowered by extremely slender models (27, 28). Among the fashion superpowers, France has been particularly affected by the cult of thinness: ‘Paris thin’ has become a concept among models and agents and refers to the excessive thinness required by the shows of the *haute couture* fashion houses (29).

The excessive emphasis on appearance and body weight, intense competition, and the prevalent use of clinically underweight models in the fashion industry may exacerbate appearance concerns among fashion models (7, 20, 30). The need to do well, to conform to industry standards, to be financially independent and to succeed at a young age can even be anxiety-forming (18).

### 4.1 Discussion of results of eating and body concerns

#### 4.1.1 BMI values

The average BMI of fashion models was 16.9 (SD= 1.60, range 14.0 – 23.7) which is classified as moderately underweight (11). Even though previous findings also confirm models being underweight, this is a considerably lower result. Previous data shows self-reported BMI values of 17.7 (26), 17.4 (13) and 17.0 (31). However, it was suggested that professional models’ perceived BMI is significantly lower than the experimenter-measured BMI (i.e., 17.0 vs. 18.6) (31). According to fashion industry standards, the bodyweight of models is insignificant, as they are judged by the measurements of their height, bust, waist and hips (17). This proposes that fashion models might be uncertain about their actual bodyweight resulting in biased BMI results (31). Models proved better at accurately estimating their body measurements and overall size compared to non-models using a three-dimensional avatar (32). Another explanation for the low BMI value could be that our study group consists of internationally recognised high fashion models, mostly working at fashion shows where size requirements are stricter than in the commercial sector, thus they might feel a stronger influence to obtain an extremely thin physique (2).

#### 4.1.2 Full or partial eating disorder symptoms

We found that 36.9% of the participants portrayed to experience EDs either manifested before their modelling career or during the modelling years, or showed very severe ED-like symptomatology, including both clinical or subclinical AN and BN. The existing data on the prevalence of EDs among fashion models is inconsistent and presents contradictory results (6). Seemingly, professional models are more prone to develop partial syndrome EDs, rather than their full-blown forms. The occurrence of subclinical AN is significantly greater among models, 14.6% (p < 0.001) compared to 2.7% of control subjects (14). Similar results were published in 2002, 12.7% of fashion models had partial AN, and an additional 7.9% had partial BN symptoms (26). In 2008, strictly similar results were found: 12.7% of professional models fulfilled partial syndrome criteria of AN, but no significant difference was shown between fashion models and control group in full-blown ED symptoms (7). Further research states that the prevalence of EDs is not higher among professional fashion models (1). The current result is notably higher. The difference could stem from the nature of questionnaire-based and interview-based evaluations. While fashion models showed no significant distinctions from their peers on self-compiled inventories, they were more inclined to acknowledge symptoms within the spectrum of EDs during face-to-face interviews (7). We must acknowledge that models were not examined by medical professionals, and self-reported diagnosis might not be accurate. It has also been suggested that women predisposed to disordered eating symptoms might gravitate towards the fashion industry (7).

#### 4.1.3 Body satisfaction

Statements about one’s body becomes very dominant amongst models. Negative body remarks were the most prominent (89.3%) in the study group. These results are in alignment with previous findings of professional models portraying higher drive for thinness and dysfunctional investment in appearance (30). Interestingly, extended time period in the modelling profession correlated with enhanced body appreciation but concurrently showed stronger drive for thinness, indicating that already underweight models have a strong desire to maintain their low body weight or become thinner (30). It is noteworthy that almost two thirds of the models (64.3%) had positive claims about their physiques in our study, but it was not correlated with the amount of time spent with modelling.

Such negative body image concerns can lead to manifesting symptoms of disordered eating (33) and diminished mental health (34). The current study shed light to some very interesting relations between certain body controlling behavior in the modelling industry, providing evidence to previous assumptions. Negative body perception was in relation with negative claims about eating, more frequent excessive calorie restriction, laxative abuse and body image disorder-like symptoms. Thus, the fashion industry has been criticized that it creates a ‘toxic’ environment being the foundation of increasing body image disorders and EDs (18, 20). This statement could be accurate, as models’ higher self-reported BMI is significantly associated with poorer body appreciation and greater body dissatisfaction (30).

However, contradicting data can also be found in the literature. No significant differences were found between models and non-models regarding body dissatisfaction (7) and body satisfaction (35). It has been proposed that fashion models may possess personality profiles enabling them to cope better with the pressures of maintaining a thin figure. Alternatively, certain aspects of the job, such as the boost to self-esteem derived from conforming to societal or industry ideals, may serve as a defense against negative body image (1). Models may recognize that their adherence to societal standards of appearance, particularly the thin ideal, could serve as a protective factor mitigating negative body image (30).

#### 4.1.4 Weight manipulating behaviors

Due to the intense pressure to maintain the size requirements of the fashion industry, a considerable number of models use weight manipulating behaviours like restricting food intake, exercising excessively, using laxatives and even self-induced vomiting (8, 13).

##### 4.1.4.1 Dieting

The current study’s participants showed some type of dietary control in 78.6% of the cases, which aligns with the previous quantitative findings. It has been previously reported that several dieting methods are at practice amongst professional models, including skipping meals (56.5%), dieting (70.5%), fasting (51.7%), or using weight loss pills (23.6%) (13). Extreme calorie restriction was used in 40.5% of the cases. In our study, 27.4% of the models portrayed monotrophic eating, and only 3.6% of the models juice fasted in order to lose weight, which is much lower than published by Rodgers et al. (13). Our finding should be understood on a general level as we did not conduct the study at specific period in the fashion industry (e.g., Fashion Weeks), but 46% of models were found to specifically lose weight for New York Fashion Week Fall’18 (10). Clean eating is also a form of dietary restrictions (36). Social standards impede the identification of orthorexia nervosa, potentially resulting in the escalation of more severe EDs as symptoms advance (37).

##### 4.1.4.2 Self-induced vomiting, laxative abuse

Besides restricting one’s calorie intake, self-induced vomiting is also applied for weight controlling purposes. Twelve participants (14.3%) used such method. Former studies show both lower (8.2%, 13; 7.5%, 8) and higher (25%, 10) frequencies for temporary self-induced vomiting. Models are more likely to experience higher levels of professional pressure during specific periods, e.g., New York Fashion Week, thus the higher results. Our examination was not conducted during such period. This further reinforces the assumption to the ED-forming nature of the fashion industry.

However, after better understanding the answers, the transcripts revealed that making oneself sick is due to previous food restriction or intentional starvation. This method usually leads to yoyo dieting and weight fluctuation (38). We believe that this result underscores the validity of our hypothesis that many of the fashion models use weight controlling methods due to external pressure, which leads to rebound effects and yoyo dieting on the long term. Weight gain was experienced in 21.4% of the participants. Severe calorie restriction results in increased urge for binge eating and additionally, in weight gain, called binge priming (39).

*“[ … ] but after a while we still gain weight and so in fact I especially imagine when you have not eaten for a very long time, when you eat, you eat again 10 times more. That’s sure yoyo. So that’s why diets are very bad but at first it works. [ … ]”* (Excerpt from transcript #87)

Laxative abuse is also a form of purging, occurrent amongst 7.4% of the participants, compared to an almost double rate found by Rodgers et al. (13%; 2017). Losing control over food intake resulted in significantly more frequent binge eating, self-induced vomiting, weight gain and ED related statements.

##### 4.1.4.3 Exercising

Exercising was favoured by models, 40.5% of the participants gave positive statements about sports activities, while only 14.3% of the models had any negative relation to exercising. For weight controlling purposes, it was found that 81.2% of the models engage in physical activities regularly and 69.4% of those models were told to “tone up” in order to book more modelling jobs (13). However, such habits can become extreme (23.8%) or even obsessive (11.9%) due to the intense pressure of the agents and designers.

The restrictive diets, excessive exercising habits, purging, consumption of laxatives, especially at younger age can cause serious health consequences. Digestive problems, hair loss, amenorrhoea, cardiac complications, hormonal imbalances, osteoporosis are all serious implications of an insufficient food intake and mannequins are at risk of such outcomes (14). Federal law was adopted in France, implying that models must obtain a health certificate from a doctor declaring that they are in good health (40), however, eating and exercising behaviours are poorly assessed hindering the intervention for EDs (10).

#### 4.1.5 External remarks about physical appearance

Our study reveals that 83.3% of the models have experienced criticism related to their appearance in the fashion environment. These negative remarks mostly target body measurements, especially hip circumference, but also extends to skin and hair condition, facial features, teeth and even clothing style. These types of negative remarks, that can potentially be considered bullying, are very dangerous during adolescence (most fashion models are still minors) as personality development hasn’t finished yet and such sentences can lead to low self-esteem, distorted self-perception, body-image disorders and potential development of EDs (41), moreover, negative weight-related remarks can be remembered for years, maintaining negative body associations (42). Such negative remarks concerning losing weight and changing body size directed to already underweight models are very frequent among fashion models (13).

Conversely, the occurrence of body appreciation from industry participants is roughly half of the frequency of body criticism, namely 44.0%. If we investigate those positive feedbacks in detail, it becomes clear they mostly appraise models’ weight loss. Models confessed that those appreciative words are usually followed by criticism or that they seem dishonest.

*“[ … ] they would praise you if it looked like you lost some weight, and they would quickly remark if by any reason you had 92 cm hips. [ … ]”* (Excerpt from transcript #56)

*“[ … ] Oh, you lost weight, you look really good, but you know, for Fashion Week you need to lose a bit more weight! [ … ]”* (Excerpt from transcript #16)

Models being negatively criticized by other industry members talked significantly more often about eating in an unfavorable manner, however, unpleasant remarks about exercising were less frequent. This finding is in alignment with the intense drive for thinness and pressure to use unhealthy weight controlling behaviors to conform to the extremely thin industry standards. Models might find comforting exercising to achieve their desired body shape and to fulfill their drive for thinness, hence the negative relation.

### 4.2 Discussion of results of other psychological aspects in fashion models

According to our findings, not only the occurrence of EDs and body image disorders seemed heightened among professional models, but also other psychological disturbances and abuse of different sources are existent in this population. The mental well-being of models is at great risk and should be better protected.

#### 4.2.1 Psychological disturbances

Due to the intense pressure models must face, the uncertain working environment, loneliness, and the continuous rejections brings a mental burden on these young women (43), that potentially manifest in different kinds of psychological disturbances. Almost half of the participants (48.8%) reported psychological difficulties throughout their modelling days. The participants mentioned in their rapport depression, severe anxiety, insecurities, low self-esteem, sudden mood swings, excessive crying, insomnia, obsessive-compulsive tendencies, body dysmorphia, and even suicidal thoughts. It is important to highlight that the participants only submitted self-narrated reports; thus, the diagnosis might not be entirely accurate. Uncertainty and the unpredictable nature of the modelling profession can play a factor in controlling one’s physical appearance, food consumption, or exercise habits (44, 45). Each fashion season is unique, and models can be replaced at any moment (17) and the frequent rejection is described as “soul-destroying” and even traumatic (18). People with different personality types react differently to external pressure, and they develop different coping mechanism to bear those stressors (46), potentially resulting in maladaptive coping mechanisms such as the manifestation of EDs or the use of illicit drugs (26).

#### 4.2.2 Abuse

One quarter of models (25.0%) mentioned in our study that they have experienced some type of emotional, psychological or physical harassment during their modelling career. Most of the time, these abuses are of emotional or psychological nature, targeting body shaming, belittling and humiliation due to models’ body measurements, mainly in front of other actors of the fashion industry. However, in our present study, agreement between the Coders was poor in terms of defining abuse (Krippendorff’s alpha = .259). Models were told 63.1% of the cases by agents that they would secure more modelling jobs if they lost weight (13), and are denied receiving financial aid if the measurement requirements are not met (18). Models are prone to be exposed to abuse of sexual nature, such as being photographed while changing backstage (10).

#### 4.2.3 Psychotherapy

A total of 16.7% of the participants reported to have engaged in some form of psychotherapy. This is considerably higher compared to the 3.5–9.9% range observed by the 2019 EHIS survey (47) and higher than the percentage observed by the National Center for Health Statistics with 9.5% of adults reporting to have received counselling or therapy (48). This is understandable if we consider the traits of professional modelling, on an international level. On the one hand, for a successful psychotherapy, frequent therapy sessions are needed, and even if those sessions are conducted online, the unpredictability of a model’s schedule and the different time zones make it difficult to adhere to a well-structured therapy program. On the other hand, it is questionable how supportive the fashion industry is towards mentally healthy models. Assertive personalities are potentially less likely to conform to such abusive and belittling environment. Having healthy bodily standards is also disadvantageous in the fashion world, as models might not meet the extreme size requirements once they adopt healthy lifestyle choices – both mentally and physically (17). The models engaging in psychotherapy mentioned more ED related content, more overeating, more self-induced vomiting and less positive remarks about eating, suggesting that fashion models mostly seek out professional help to treat ED symptoms. However, they made fewer negative remarks about their bodies, more about weight gain and less body image disorder symptoms which envisions that they were able to overcome bodily concerns and be more acceptive of themselves.

### 4.3 Extrinsic or intrinsic pressure?

It has been a debated question whether young women with already existing EDs or ED like tendencies chose to pursue modelling as it is an acceptable lifestyle to validate their illness or if models start to manifest disordered eating habits and body image problems due to the external stressors (7). Considering all the above mentioned, the latter seems more accurate. It appears probable that fashion models manifest increased ED and body image concerns due to intense environmental pressure deriving from the fashion industry. This assumption might be further justified if we consider that agents prefer to choose young women with very slim, almost anorexic-like body frames to sign modelling contracts (49). It is questionable whether those girls possess unique natural body constitutions within genetic variability (35) or must take tedious efforts to maintain such measurements (13). Fashion models with higher BMI values show more ED symptoms (31) implying that aspiring models with normal body constructs engage in extreme weight loss methods to meet industry standards. Our overall interpretation of the data is that fashion models experience immense environmental pressure to conform to the extremely slim beauty ideal.

To our best knowledge, the current study involved a larger number of multicultural female fashion models than any previous qualitative research on the field. Furthermore, this is the first ever study to use thematic content analysis for the assessment of ED-like symptoms and body image disturbances in this population, thus we believe it makes a significant contribution to the existing literature.

Certain limitations must be considered regarding this study. Firstly, most of the respondents were not native English speaker which could cause discrepancy due to misunderstandings or in the ability to express complex ideas. However, the level of linguistic complexity in the testimonials contradict this assumption. Secondly, some of the participants were recruited during the COVID-19 pandemic, when less modelling jobs were available which could also alter their responses. Furthermore, the self-reported anthropometric data may differ from the factual values, so the calculation of BMI may be distorted (50). Thirdly, answers to sensitive questions may have been biased as we assume that those models participated in our study who are more active in creating change in the fashion industry and who disagree with current dynamics. At the same time, it is also possible that models decided to hide certain details in the hope of protecting their career, even though complete anonymity was ensured. Furthermore, underestimation of the symptoms might be possible not only due to career-protection, but also due to the nature of the questions, as they were not explicitly asked from the participants. Models were not examined by health care professionals, and self-reported diagnosis might not be accurate. The first author’s personal experience as a former fashion model may introduce an inherent bias to this research, potentially influencing the framing of questions, interpretation of responses, and overall analysis, despite efforts to maintain objectivity throughout the study. Lastly, we have to mention that hand-coding a big number of lengthy testimonials is an exceptionally strenuous task, challenging the most experienced coders as well. The enduring efforts might have caused discrepancies.

## 5 Conclusion

The fashion industry plays a vital role in shaping cultural ideals. Based on the personal testimonies of fashion models, it can be concluded that there is a significant risk for developing EDs within this specific population, either in clinically severe forms or more often, manifested as subclinical symptoms. These phenomena can be occurrent due to the intense environmental pressure towards maintaining a slim physique. The physical and mental health of models must be taken into consideration when defining beauty standards of the fashion industry. There is a need to revise unrealistic measurement requirements, and agencies should refrain from pressuring models into engaging in such health-damaging behaviours to conform industry standards.

Further studies are required to investigate the risk factors and the actual frequency of EDs and body image disorders among professional fashion models. The symptoms assessed should go beyond AN and BN. The consequences of the environmental pressure can highly influence the mental health status of the fashion models. Emotional or sexual abuse, exploitation and humiliation are also parts of the fashion industry. The results hold significant relevance in shaping health regulations within the fashion industry to curb the prevalence of EDs among models and individuals exposed to model imagery. It is imperative for the fashion industry to implement alterations that prioritize the physical and mental well-being of fashion models and to reconceive existing regulation relying on BMI values as broader mental and physical aspects should be evaluated for effective preventive measures against eating and body image disorders amongst fashion models. This entails discontinuing the promotion of health-detrimental practices enforced by agents or designers and establishing a system of regular medical consultations.

## Data availability statement

The raw data supporting the conclusions of this article will be made available by the authors, without undue reservation.

## Ethics statement

The studies involving humans were approved by Regional Research Ethical Board of the Semmelweis University Budapest (No. 3/2020). The studies were conducted in accordance with the local legislation and institutional requirements. Written informed consent for participation in this study was provided by the participants’ legal guardians/next of kin.

## Author contributions

NB: Conceptualization, Data curation, Investigation, Writing – original draft, Writing – review & editing. PK: Formal analysis, Methodology, Writing – review & editing. FT: Supervision, Writing – review & editing.

## Supplementary material

Supplementary Material 1 Series of open questions used for the present study.
