# Supplementary material for: Increased eating disorder frequency and body image disturbance among fashion models due to intense environmental pressure: a content analysis
Source: Front Psychiatry. 2024 Apr 3;15:1360962. doi: 10.3389/fpsyt.2024.1360962 (PMC11021945; doi:10.3389/fpsyt.2024.1360962)
Supplement: Supplementary Material 1 — Series of open questions used for the present study. [file DataSheet_1.docx]

**Supplement 1**

**Series of open questions used for the present study**

1. What did you think about modelling as a child? How did you get started?
2. Did you feel that you had to meet certain requirements? If so, did you have to follow a diet or an exercise routine to meet the requirements?
3. Can you briefly sketch your modelling history?
4. Did you feel more pressure about your weight after you signed with an agency?
5. What kind of relationship did you have with your agents? How did you feel about them?
6. Did they encourage you to lose weight/get in shape?
7. What do you think about the size requirements of the fashion industry, in general? How difficult is it to reach and maintain these requirements?
8. Would you like to see changes in the image represented by the industry?
9. How do you maintain your physique? What are your eating habits and how much exercise do you do? How have these habits changed over the years? (please think about the diet changes prior to Fashion Week or an important shooting as well)
10. How did Fashion Week affect you both mentally and physically?
11. Have you experienced any fluctuations in your weight throughout the years?
12. Have you ever felt that the requirements of the fashion industry have a positive or negative impact on your mental or physical health?
13. How have you felt about yourself and your body, both your physical appearance and inner values in general, have you experienced insecurities or judgement?
14. Have you ever worried about your physical appearance?
15. How did your agents or other industry members make you feel about yourself?
16. Why do you think designers represent their collection with size 0 models?
17. Have you found any difference in the size requirements of different countries?
18. Do you consider other girls your competition?
19. Who is your beauty ideal?
20. What changes would you like to see in the fashion industry?
21. What do you think about curve size and plus size modelling?
22. How do you feel about the industry recently? Do you see any changes?
23. What are the positive aspects of modelling? Can you share some experience that brought you joy during your career?
